# A high throughput live transparent animal bioassay to identify non-toxic small molecules or genes that regulate vertebrate fat metabolism for obesity drug development

**DOI:** 10.1186/1743-7075-5-23

**Published:** 2008-08-27

**Authors:** Kevin S Jones, Alexander P Alimov, Horacio L Rilo, Ronald J Jandacek, Laura A Woollett, W Todd Penberthy

**Affiliations:** 1 Department of Genome Science, University of Cincinnati, Cincinnati, OH, 4523, USA; 2 Department of Surgery, University of Cincinnati, Cincinnati, OH, 4523, USA; 3 Department of Pathology and Laboratory Medicine, University of Cincinnati, Cincinnati, OH, 4523, USA

## Abstract

**Background:**

The alarming rise in the obesity epidemic and growing concern for the pathologic consequences of the metabolic syndrome warrant great need for development of obesity-related pharmacotherapeutics. The search for such therapeutics is severely limited by the slow throughput of animal models of obesity. Amenable to placement into a 96 well plate, zebrafish larvae have emerged as one of the highest throughput vertebrate model organisms for performing small molecule screens. A method for visually identifying non-toxic molecular effectors of fat metabolism using a live transparent vertebrate was developed. Given that increased levels of nicotinamide adenine dinucleotide (NAD) via deletion of CD38 have been shown to prevent high fat diet induced obesity in mice in a SIRT-1 dependent fashion we explored the possibility of directly applying NAD to zebrafish.

**Methods:**

Zebrafish larvae were incubated with daily refreshing of nile red containing media starting from a developmental stage of equivalent fat content among siblings (3 days post-fertilization, dpf) and continuing with daily refreshing until 7 dpf.

**Results:**

PPAR activators, beta-adrenergic agonists, SIRT-1 activators, and nicotinic acid treatment all caused predicted changes in fat, cholesterol, and gene expression consistent with a high degree of evolutionary conservation of fat metabolism signal transduction extending from man to zebrafish larvae. All changes in fat content were visually quantifiable in a relative fashion using live zebrafish larvae nile red fluorescence microscopy. Resveratrol treatment caused the greatest and most consistent loss of fat content. The resveratrol tetramer Vaticanol B caused loss of fat equivalent in potency to resveratrol alone. Significantly, the direct administration of NAD decreased fat content in zebrafish. Results from knockdown of a zebrafish G-PCR ortholog previously determined to decrease fat content in *C. elegans *support that future GPR142 antagonists may be effective non-toxic anti-obesity therapeutics.

**Conclusion:**

Owing to the apparently high level of evolutionary conservation of signal transduction pathways regulating lipid metabolism, the zebrafish can be useful for identifying non-toxic small molecules or pharmacological target gene products for developing molecular therapeutics for treating clinical obesity. Our results support the promising potential in applying NAD or resveratrol where the underlying target protein likely involves Sirtuin family member proteins. Furthermore data supports future studies focused on determining whether there is a high concentration window for resveratrol that is effective and non-toxic in high fat obesity murine models.

## Background

Obesity has been increasing in every state in the nation [1] and has reached an all time high in children [2]. Due to the adverse health affects of obesity the total cost for medical care and disability associated with obesity was estimated to be 99.2 billion dollars in 1995 [3]. Currently there are only two pharmaceutical drugs used for the treatment of obesity and these drugs have not been demonstrated to result in weight loss beyond 5 years [4]. Much more successful results have been obtained by performing bariatric surgery, however there are significant risks associated with these procedures. The isolation of a small molecule that can activate lipolysis could have therapeutic potential for the treatment of obesity. By extension there is a great need to identify which genes regulate fat metabolism to identify potential mechanisms of targeted molecular medicine.

Several monogenic obesity genes have been cloned in mice including leptin, leptin receptor, carboxypeptidase, melanocortin-4 receptor, and the orexigenic agouti protein (for a review see [5]). Mutations in melanocortin-4 receptor have been observed more than any other monogenic obesity disease at 1–6% of the surveyed obese population. A variety of other genes have been shown to be associated with the obesity phenotype including: UCP1-3, PPARγ, and several adrenergic receptors. A proline to alanine at position 12 of PPARγ has clearly been shown to have effects on lipid metabolism. The number of genes expected to be involved in obesity remains to be determined.

The zebrafish model organism *Danio rerio *is currently the simplest model organism complete with the full complement of vertebrate organs that can be used in forward genetic screens. ENU mutagenesis screens have been performed to isolate mutant zebrafish with defects in their temperature maintenance in an attempt to identify genes potentially involved in regulating lipid metabolism [6]. This was based in part on the observation that mice missing the leptin protein or receptor have problems regulating their temperature. However, to date no one has completed a forward chemical mutagenesis screen to result in the identification of genes regulating fat metabolism using zebrafish.

The yolk sac is maternally derived and represents the sole source of energy for the embryo and larva during early zebrafish development. Significantly, the yolk sac is a quantifiably finite source of energy that is largely consumed during the first week of larval development. These characteristics give the yolk sac distinct advantages for assaying changes in organismal lipid metabolism.

## Methods

### Materials

Zebrafish were obtained from the Zebrafish International Resource Center. All small molecules were obtained from Sigma chemicals.

### Maintenance of Zebrafish

Adult and embryonic zebrafish were maintained according to protocols described in The Zebrafish Book [7].

### Measurement of Total Fatty Acids and Cholesterol

Pools of 30 to 50 healthy 3 dpf larvae were selected for daily incubations with fresh E3 media containing small molecules until 7 dpf before they were clarified by centrifugation at 11,000 rcf for 3 min. Supernatant was removed and larvae were frozen for chemical analysis. The pool of larvae was subjected to saponification, conversion to methyl esters, and extraction with hexane. A known mass of heptadecanoic acid was added to the larvae pool before saponification. The methyl esters were quantified by gas chromatography and the mass was calculated from the ratio of the area of methyl heptadecanoate to those of other methyl esters. The conditions for the gas chromatograph have been described previously [8]. For cholesterol measurements at least 30 zebrafish were treated with 1 mM of nicotinic acid or nicotinamide from 3 to 10 dpf before extraction and quantitation of total cholesterol [9].

### Transcript Quantitation by Q-RT-PCR

Twenty 6 dpf zebrafish larvae were exposed to molecules for 11 hr and flash frozen with liquid nitrogen. RNA was quickly isolated using Qiagen RNeasy mammalian tissue protocol, incorporating Qiashredder. RNA levels were quantified using Nanodrop spectrophotometer and cDNA was synthesized using equivalent amounts of RNA using Superscript II Reverse Transcriptase (Invitrogen). cDNA was quantitated in triplicate by real-time PCR. Primers pairs used were for GPR109a (also known as HM74a, TTTCGACGCTCCCATTCTGGATGA and AGGACGAACTCGCTGAACAGAACA for 60 bp) CD36, AGATGGTTCCTCTTTCCACCCGTT and ACAGGCAGCAAGTACCGATACACA for 144 bp), FABP4 (TGAGCAGGGCGTCATCACTATGAA and TTGTGGTCTTTCCTTCCCAGGTCT for 176 bp), and these were normalized to PP1A, AGAATTTCAGGCAGTTGTGCACGG and TGTGGTTTGTGAAGTCACCTCCCT Qiagen Hot Start DNA Polymerase was used for PCR: 15 m 95°C, 45 × (25 s 94°C, 25 s 60°C, 25 s 68°C), and hold 10°C. All reaction single product sizes were first verified by visualization on agarose gels and melting curve analysis. Fluorescein was included in the reaction to provide a stable fluorescence baseline. Data was collected with the Bio-Rad iCycler iQ system using SYBR green to detect amplicon production. Analysis was performed by the comparative Ct method so both treated and untreated samples were normalized to housekeeping gene product peptidyl-prolyl isomerase A (PPIA) as an internal control.

### Hypoxic stress to Zebrafish larvae

Multi-well 48 well plates with 0.5 mL of zebrafish embryo (E3; [7]) were preconditioned to anoxic conditions by placement into a Bio-Bag type A environmental chamber (Becton Dickinson) as described previously [10]. Using a plate containing at least 20 of 6 dpf zebrafish, the water was removed and replaced with preconditioned hypoxic E3 before initiating a second Bio-Bag type A environmental chamber reaction. Zebrafish were visually monitored for lack of response to shaking stimuli. After approximately 2 hours, zebrafish were harvested by rapidly removing solution and flash freezing in tubes with liquid nitrogen for Q-RT-PCR analysis.

### Small Molecule Treatments with Nile Red and Fluorescence Microscopy

Embryos were raised in embryo 3 media (E3: 5 mM NaCl, 0.17 mM KCl, 0.33 mM CaCl2, 0.33 mM MgSO4, 10E-5 methylene blue) supplemented with 200 μM PTU [7]. Starting at 3 days post-fertilization (dpf), 30 to 50 healthy larvae were placed into 48 well plates containing E3 with 10 mM Tris pH 7.4 and 10 ng/mL nile red at a density of 10 larvae per well. Nile red was dissolved in 50,000 × stock solution at 500 μg/mL in acetone. In order to quantitatively prepare the nile red incubation solution 10 μL of stock solution was first carefully pipetted into 50 mL E3 media to prepare a 10× solution. This was diluted to a final working concentration of 10 ng/mL. Incubating larvae at higher levels of nile red produced a bright signal throughout the larvae. Incubation times ranged from 6 hours to overnight to allow for the nile red to equilibrate and penetrate into the depths of the yolk sac and central nervous system of 7 dpf larvae as monitored by fluorescent microscopy. Incubations were performed without shaking at 28°C. Media was replaced daily until 7 dpf by transferring larvae to plates containing solutions pre-heated to 28°C using baskets. To first determine the working concentration, groups of 3 dpf zebrafish were exposed to a ten-fold serial dilution of small molecules and allowed to develop. Ten-fold sub-lethal dose of molecules was chosen as the working concentrations for experiments. Molecules were refreshed on a daily basis starting at 3 dpf. Media was supplemented with 100 μM nicotinic acid, 100 μM nicotinamide, 50 μM resveratrol (10 mM stock dissolved in DMSO), or 50 μM resveratrol with 100 μM norepinephrine. At 7 dpf larvae were examined by fluorescence microscopy. Nile red was visualized on a Zeiss Axioplan microscope using a Texas Red filter. Camera exposure time was established using untreated larva for signal normalization. Larvae to be compared were placed in a single depression slide so that the media and depth were consistent.

### Morpholino Injection

At least 30 embryos were injected with 2 nL of 0.3 mM (0.6 pmol) anti-sense morpholino per protocols for each of three separate experiments as described previously [11]. Morpholinos were designed to target the zebrafish ortholog to a G-protein coupled receptor the loss of function of which has previously been shown to result in a decrease in fat in *C. elegans *screen [12]. The *C. elegans *GenePair corresponds to locus tag H09F14.1. The zebrafish ortholog corresponds to mRNA [accession XM_001340648], which is GPR142a. A morpholino with sequence CGGGCGTCGTGCCATTGTGCCAGTC was used to target the GPR142a mRNA. As a negative control, three unrelated morpholinos were injected targeting the ortholog to B3 (TATACGAAAATGAGCGACCGTGTTG) and heat shock protein. From 3 to 7 dpf zebrafish larvae were incubated with daily refreshing of nile red before relative quantification by fluorescence microscopy.

## Results

### Nile Red Can Penetrate the Deep Tissues of 7 dpf Zebrafish Larvae to Produce a Specific Fluorescent Signal Restricted to Lipid Rich Tissues Without Exerting Toxic Effects

**Figure 1:**
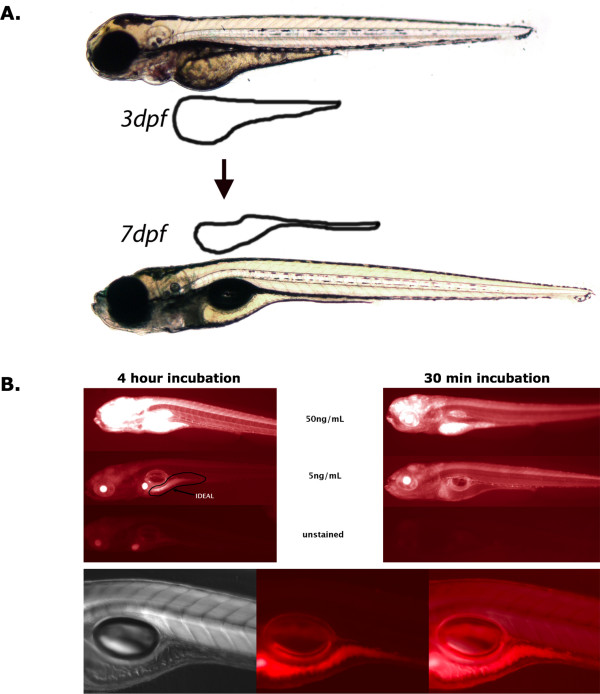
**A large portion of the yolk sac is absorbed during this sensitive developmental time window used to detect small molecule-mediated changes in fat metabolism (A).** Extended incubation at 5 ng/mL nile red provides ideal signal to noise ratio for detection of anatomically localized fat stores (B). Overnight incubation was even better with the signal in the gall bladder often disappearing (shown in Fig. 4).

Early development of the zebrafish proceeds quickly to insure greater chance for survival in nature. By just 3 dpf the protruding mouth has been formed. Thus oral delivery of small molecules can be taken into consideration by this stage of development. At 3 dpf zebrafish siblings generally have equivalent amounts of lipid content, thus providing a good baseline for quantifying subsequent changes in lipid metabolism. Volume measurements reveal that over 50% of the volume of the yolk sac is depleted for development from 4 to 7 dpf (Fig 1A. [13]). For these reasons we believe this developmental time window is particularly ideal for providing a potentially sensitive read out of fat metabolic activity identify molecular activities regulating vertebrate animal fat metabolism.

To establish the staining protocol, a 10 fold series of concentrations of nile red were tested starting from the established concentration used previously in *C. elegans *genetic screens [12]. A concentration of 10 ng/mL final nile red concentration was ideal for obtaining a signal significantly greater than background yet not present throughout the entire embryo. The bulk of signal was restricted to the lipid rich yolk sac (Fig. 1B). No overt nile red associated toxicity or effects on viability were observed in zebrafish. Groups of 6 dpf zebrafish were incubated with nile red for times ranging from 30 min to overnight. We determined that at least 8 hours were needed to detect signal in the deeper regions throughout the embryo.

### Pharmacological Targeting of PPARγ, SIRT-1, beta-adrenergic receptors, and GPR109a is Generally Evolutionarily Conserved from 7 dpf Zebrafish Larvae to Man

**Figure 2:**
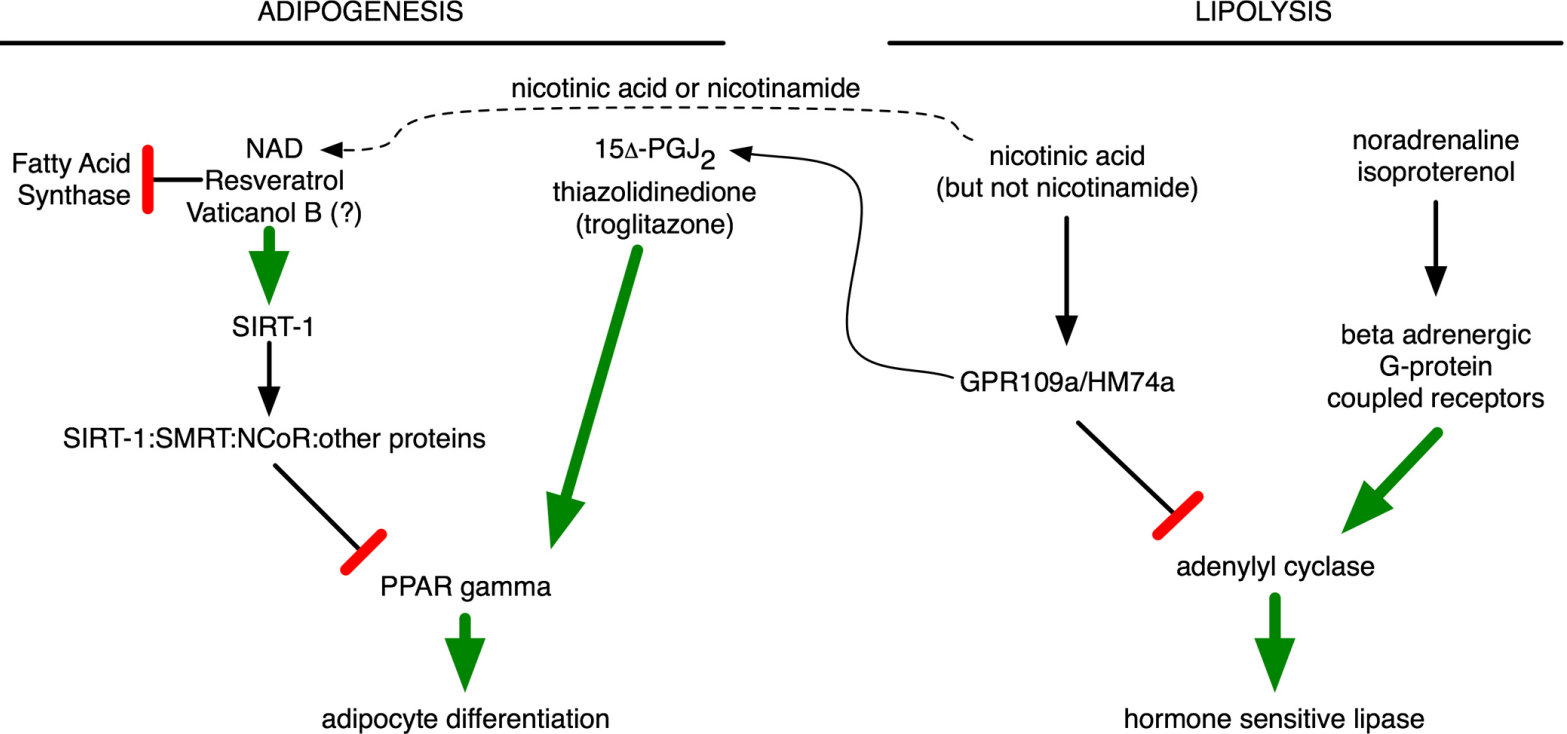
**Pathways regulating PPARγ-mediated adipocyte differentiation are shown on the left, while G-PCR targeted pathways working via cAMP-mediated signal transduction to regulate lipolysis are shown on the right**. The pharmacologically targeted G-PCRs involved in lipolysis are GPR109a/HM74a or beta-adrenergic receptors. Nicotinic acid similarly affects both processes, inhibiting lipolysis and promoting adipocyte differentiation through PPARγ activation. However, nicotinic acid is also a precursor to NAD, which exerts the opposite effect (left). Resveratrol can both directly inhibit fatty acid synthase and cause activation of SIRT-1 activity, both of which are known to decrease fat content in adipocytes. Vaticanol B is an only just recently characterized tropical tree bark-derived resveratrol tetramer determined to have much greater anti-inflammatory activity than resveratrol. Thus, we predict that Vaticanol B may be similarly involved in controlling fat metabolism.

To be of greatest clinical relevance the zebrafish animal model should be responsive to known mammalian pharmacology regulating fat metabolism where both increases or decreases in fat content should be detectable under the assay conditions. To examine zebrafish pharmacology and assay sensitivity, we targeted PPARγ or the hormone responsive lipase (Fig. 2). Then total fat, total cholesterol and gene expression of markers of differentiation were quantified.

**Figure 3:**
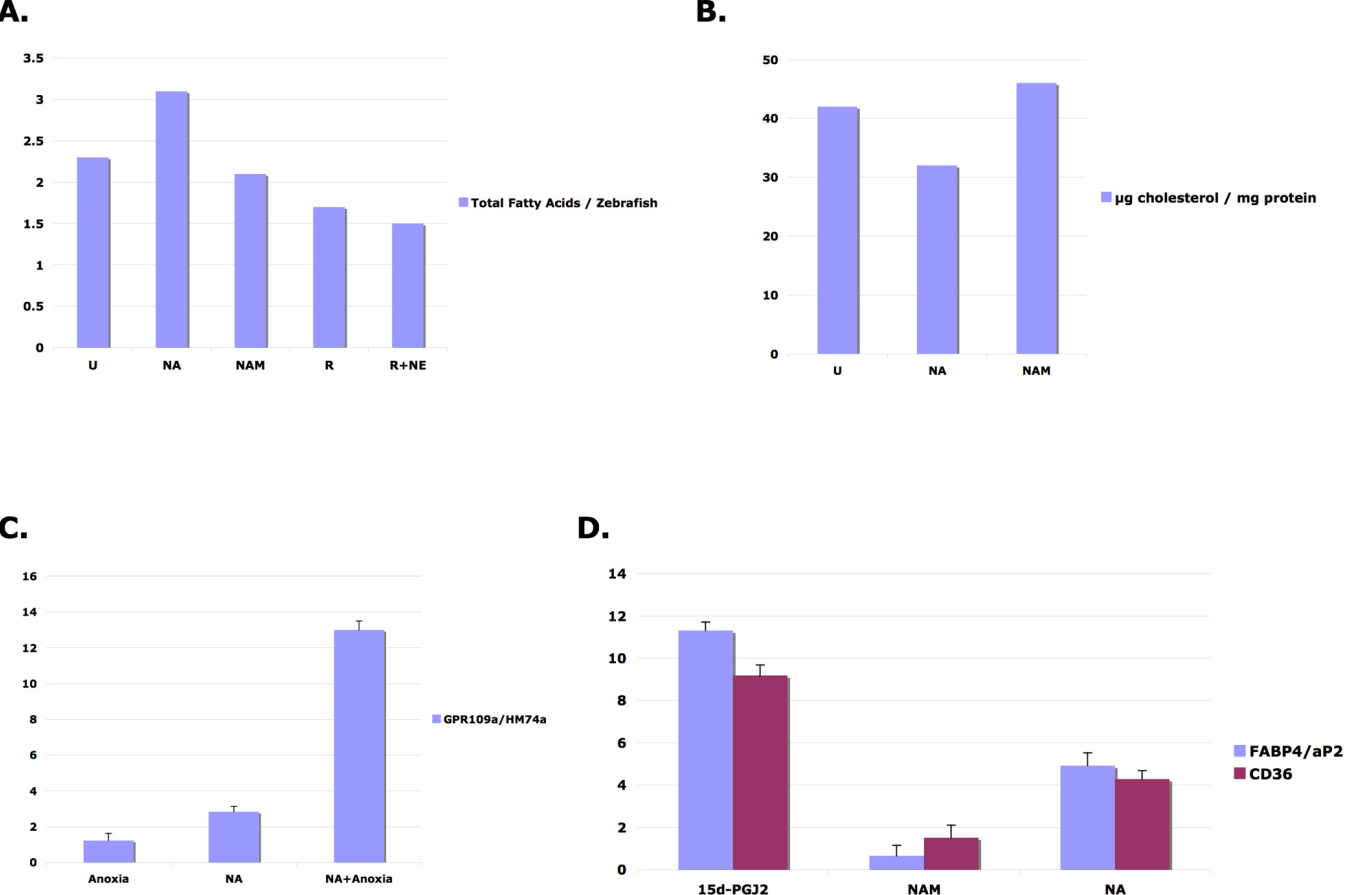
**Zebrafish respond to mammalian regulators of fat metabolism in an evolutionarily conserved manner**. Similar to pharmacological application of niacin in the clinic, nicotinic acid inhibits zebrafish lipolysis (A), decreases total cholesterol (B), and increases expression of the most potent quantitative scavenger of oxidized lipids, CD36 and the classic marker of adipocyte differentiation, FABP4 (D). Zebrafish larvae were exposed to nicotinic acid (NA), nicotinamide (NAM), resveratrol (R), or resveratrol with norepinephrine (NE) from 3–7 dpf before extraction of total phospholipids for quantitation by gas chromatography (A) or quantitation of transcript levels (C and D). For cholesterol measurements zebrafish were treated with 1 mM concentrations of NA or NAM from 3–10 dpf (B).

Insulin is the most potent known physiological inhibitor of lipolysis, while nicotinic acid is a potent pharmacological inhibitor of lipolysis [14]. Treatment of zebrafish larvae from 3 to 7 dpf with 100 μM nicotinic acid but not nicotinamide resulted in detectable increases in total fat, whereas treatment with 100 μM resveratrol caused significant decreases in detectable fat (Fig. 3A). This decrease in fat was even further decreased when resveratrol was supplemented with 20 μM norepinephrine consistent with previous results performed using murine adipocytes [15]. Treatment with 1 mM nicotinic acid from 3 to 10 dpf also caused a significant 24% decrease in total cholesterol from 42 ng cholesterol per mg protein to 32 ng/mg (Fig. 3B). Nicotinamide did not significantly alter the total cholesterol (46 ng/mg). These results are consistent with the long known ability of nicotinic acid but not nicotinamide to decrease total cholesterol in the clinic.

Even though nicotinic acid has been used in the clinic for over 50 years to treat high cholesterol, it was only recently that the nicotinic acid G-protein coupled receptor was identified [16], thus elucidating a critical link in the mechanism of action for one of the most effective drugs known to prevent cardiac mortality [17]. This receptor has also been identified as a gene up-regulated in response to hypoxia or interferon gamma treatment in macrophages [18]. The closely related molecule nicotinamide, does not bind to the high affinity nicotinic acid receptor with any appreciable affinity, but like nicotinic acid it does provide NAD (Fig. 2). Thus nicotinamide is a negative control for nicotinic acid signal transduction through the high affinity nicotinic acid G-PCR GPR109a. Nicotinic acid treatment to activate GPR109a caused the total fat content of zebrafish larvae to increase, while nicotinamide did not exert any dramatic change in total fat content (Fig. 3A). Thus, nicotinic acid responsive anti-lipolytic activity is present in 6 dpf zebrafish similar to mammals.

Since the high affinity nicotinic acid G-protein coupled receptor is of such great clinical significance we examined the transcriptional regulation of GPR109a/HM74a after nicotinic acid treatment and hypoxic stress by quantitative RT-PCR. Hypoxic stress applied to 6 dpf zebrafish larvae caused induction of GPR109a transcription (Fig. 3B) consistent with previous observations linking hypoxia mediated up-regulation GPR109a in monocytes [18]. Treatment with nicotinic acid by itself caused an increase in GPR109a transcription that was synergistically enhanced by hypoxia treatment, thus suggesting a positive feedback loop (Fig. 3C).

To determine whether zebrafish PPARγ is responsive to classical PPARγ activators, we exposed 6 dpf zebrafish to 20 nM 15d-PGJ_2 _overnight. The prostaglandin 15d-PGJ_2 _is the most potent known endogenous activator of PPARγ. Adipocyte fatty acid-binding protein (FABP4) and CD36 are classic PPARγ target genes in mammals [19]. Treatment of zebrafish larvae with 15d-PGJ_2 _overnight caused a greater than 10 fold increase in the amount of FABP4 and CD36 transcripts in zebrafish similar to what has been observed in mammals (Fig. 3D). Nicotinic acid activation of GPR109a leads to localized endogenous 15d-PGJ_2 _biosynthesis at professional antigen presenting cells (Langerhans dendritic cells and macrophages, of which there are many in 6 dpf zebrafish larvae), which similarly activates PPARγ [18] leading to increased transcription of FABP4 (also known as aP2) and CD36 target genes in mammals (Fig. 2). Treatment of zebrafish with nicotinic acid resulted in increased gene expression of FABP4 and CD36, while treatment with the related NAD precursor nicotinamide did not cause any appreciable change. Together these results are consistent with a high degree of evolutionary conservation in zebrafish related to mammals for PPARγ (15d-PGJ_2 _and nicotinic acid), SIRT-1 (resveratrol), beta-adrenergic receptor (norepinephrine, isoproterenol), or GPR109a (nicotinic acid but not nicotinamide) pharmacology as related to fat metabolism (Fig. 2).

### Small Molecules Regulating Fat Metabolism are Visually Detectable in Live Zebrafish larvae

**Figure 4:**
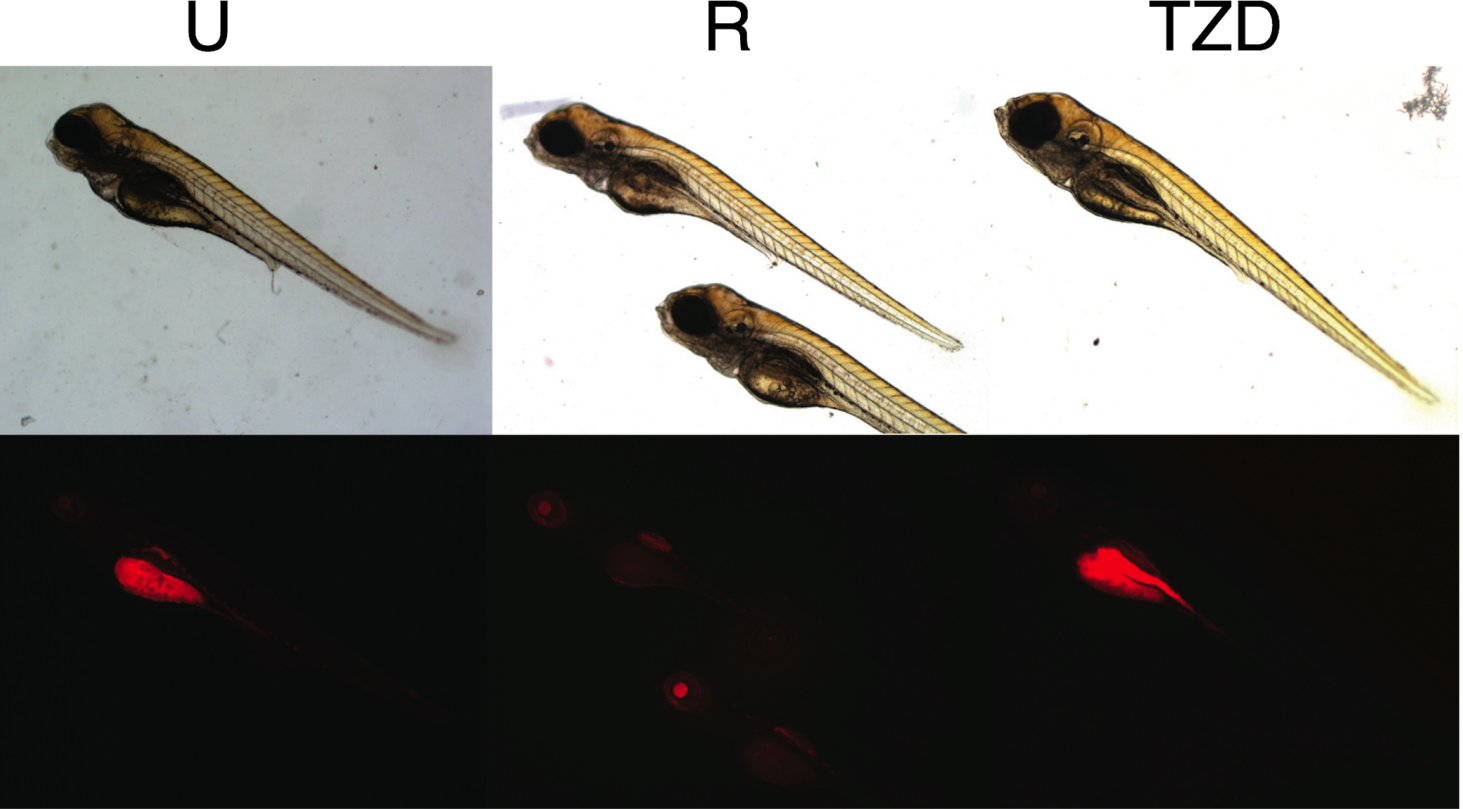
**Pharmacology causing decreases or increases in fat metabolism is visually detectable in a quantitative fashion by using live zebrafish nile red fluorescence microscopy.** Zebrafish were incubated with daily refreshing of fish water containing nile red with or without 100 μM resveratrol (R) or 10 nM troglitazone (TZD). Exposure time for all fluorescent images was set to that of untreated zebrafish (left). Bright field images are provided to show lack of overt toxicity.

**Figure 5:**
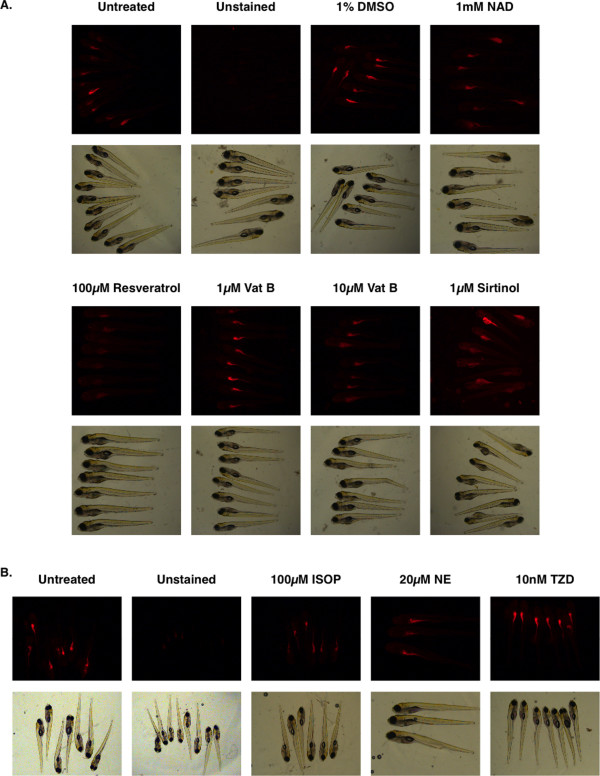
**Conservation of pharmacological pathways and consistency amongst groups of animals are shown**. NAD or resveratrol is known to activate SIRT-1. Conversely, sirtinol is an inhibitor of SIRT-1. Vaticanol B (Vat B) is a resveratrol tetramer natural product predicted to activate SIRT-1 as well (A). Isoproterenol or norepinephrine activation of beta-adrenergic receptors causes a decrease in detectable fat. Conversely, activation of PPARγ promotes adipocyte diferentation as predicted for treated zebrafish larvae. This slows the normal reduction in fat stores seen during this window of development.

To determine whether we could visually detect both increases and decreases in this whole animal assay of fat metabolism we incubated zebrafish larvae in nile red containing embryo media and captured fluorescence microscopy images at 7 dpf. Exposure time used for capturing all images were normalized to that initially obtained by automatic exposure of untreated stained solvent only zebrafish larvae. For determining whether we could detect pharmacological decreases in fat we used activators of the beta-adrenergic receptor (isoproterenol or noradrenaline) or SIRT-1 (nicotinamide adenine dinucleotide, resveratrol, or potentially Vaticanol B). To determine whether we could visually detect increases in fat we used specific activators of PPARγ (the thiazolidinedione drug troglitazone or 15d-PGJ_2_, Fig. 2). Resveratrol treatment resulted in highly reproducibly detectable decreases in fat. Conversely, treatment with just 20 nM of the highly stable PPARγ activator troglitazone resulted in visually detectable increases in fat (Fig. 4 and 5). Treatment with the SIRT-1 inhibitor sirtinol was less consistent in results due to poor solubility combined with randomness of larval movement activity. Nonetheless, some fish did exhibit increased detectable fat.

Several tetramers of resveratrol derived from acetone extracts of tree bark have garnered increased attention owing to their ability to inhibit the map kinase pathway and thus suppress cancer cell line growth. Vaticanol B in particular was distinctively more active than resveratrol in its ability to inhibit LPS-mediated production of nitric oxide, TNFα, and PGE_2 _[20]. However, it is not known how this compound affects fat metabolism. Thus we examined fat metabolism using the zebrafish larvae nile red fluorescence assay. Treatment of zebrafish with Vaticanol B revealed a fat reducing activity that was approximately equivalent in potency to that of resveratrol itself (Fig. 4A). Targeting of the beta-adrenergic receptor with norepinephrine or the more chemically stable molecule isoproterenol also resulted in reproducibly detectable decreases in fat content (Fig. 4B). All examined pharmacological data support the notion that signal transduction regulating fat and cholesterol metabolism through beta-adrenergic receptors, SIRT-1, and the high affinity nicotinic acid G-protein coupled receptor GPR109a are highly evolutionarily conserved from fish to man. Significantly, whole zebrafish nile red fluorescence microscopy also enables examination of overt pharmacological toxicity.

### Anti-sense knockdown of zebrafish ortholog to G-protein coupled receptor previously identified in C. elegans nile red fat metabolism screen, reveals that GPR142 may be a good target of future obesity drug development

**Figure 6:**
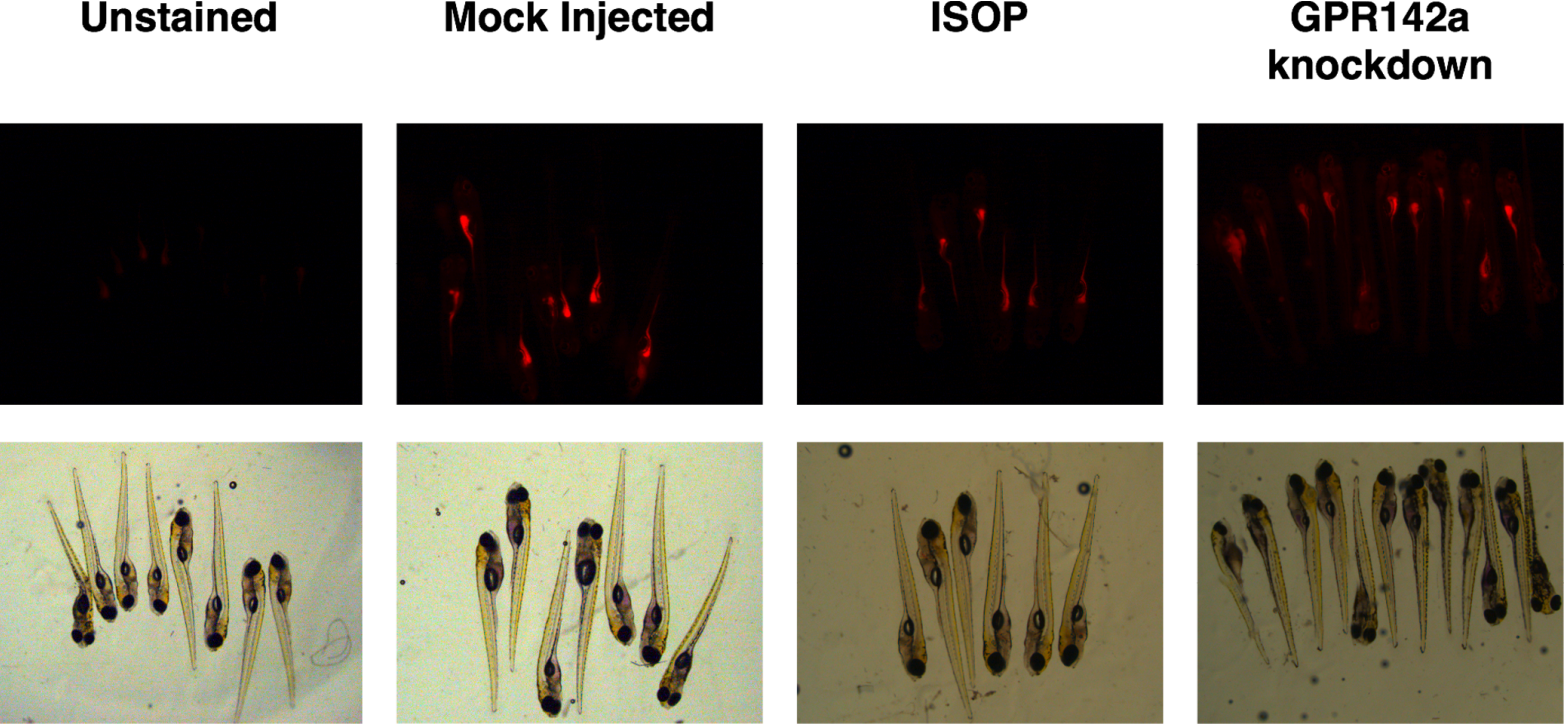
**Antagonists of GPR142 are candidate future obesity drugs**. Anti-sense knockdown of G-protein coupled receptor gene products the loss of function previously determined to cause decrease in fat content [12]. Incubation with the stable beta-adrenergic agonist isoproterenol is shown as a positive control causing increased lipolysis for comparison.

Genetic screens have previously been used to identify 305 out of 16,757 screened genes the loss of function of which results in decreased fat content in *C. elegans *[12]. From this screen we selected two G-protein coupled receptors to examine by anti-sense knockdown in zebrafish given their drug accessibility. We limited our choices to G-PCRs since they are the most commercially successful class of pharmaceutical drug targets. One G-PCR was chosen because the *C. elegans *loss of function results in increased fat content (*C. elegans *H09F14.1) while the other leads to decreases in fat (*C. elegans *F56B6.5). Knockdown of the zebrafish ortholog to *C. elegans *F56B6.5 resulted in developmental defects thus suggesting that complete antagonism of this GPCR may be toxic. Knockdown of H09F14.1 however, did cause detectable decreases in fat (Fig. 6). The zebrafish gene corresponds to GPR142a. Thus GPR142a is a candidate drug target for development of anti-obesity therapeutics.

## Conclusion

The zebrafish yolk sac is a finite maternally derived energy source that predominately dwindles in size during the first week of development (Fig. 1). In this study we determine that the rate by which the zebrafish yolk sac diminishes is predictably affected by small molecules known to regulate mammalian fat metabolism via PPARγ adipogenic and hormone sensitive lipolytic pathways (Fig. 2 and 3). Both direct (15d-PGJ_2_) and indirect (nicotinic acid) PPARγ agonists caused increased FABP4 and CD36 gene expression in 7 dpf zebrafish larvae (Figure 3D). Conversely, beta-adrenergic agonist exposure (norepinephrine or isoproterenol) led to decreases in detectable fat in zebrafish larvae (Fig. 3A and 5B).

Our examination of nicotinic acid pharmacology in zebrafish larvae also supports a high degree of evolutionary conservation for GPR109a signaling. Nicotinic acid functions as a potent inhibitor of adipocyte lipolysis by signaling through the high affinity nicotinic acid G-protein coupled receptor GPR109a. This causes Gi-coupled decreases in cyclic AMP Protein kinase A, which inhibits hormone sensitive lipase (Fig. 2[14,16]). Accordingly, zebrafish larvae exposed to nicotinic acid from 3 to 7 dpf have resultant increases in total fat (Fig. 3A). Zebrafish exposed to nicotinic acid from 3 to 10 dpf dramatically decreased (24% less, Fig. 3B) levels of total cholesterol roughly similar to that observed for patients treated for dyslipidemia with pharmacological niacin administration, which typically results in a decrease in total cholesterol of 4–16% [21]. This result supports that this window of zebrafish development is particularly sensitive to effectors of cholesterol metabolism and that zebrafish may be useful for the development of potential drugs to treat dyslipidemia that work through GPR109a.

While changes in fat content are visually quantifiable under these *in situ *whole zebrafish larvae conditions, this assay is limited by drug delivery. Our experiments using the only OTC anti-obesity drug Orlistat (also known as Alli or Xenical) failed to detect changes in zebrafish larvae fat content due to water insolubility (unpublished observations). For testing molecules with poor solubility in zebrafish it is necessary to inject them. This makes the zebrafish assay not as high throughput, but it does work and an examination of the logP of octanol:water partitioning can generally predict whether injection will be necessary [22].

Of all the molecules tested here, we see the most consistently dramatic effects in reducing nile red detectable fat after treatment with 100 μM resveratrol (Fig. 5). Resveratrol has been shown to increase mitochondrial biogenesis and to shift the health of mice fed high calorie diets to that of mice fed standard diets, significantly increasing their lifespan and motor function [23]. Resveratrol can also function as a direct inhibitor of fatty acid synthase [24]. Thus resveratrol is being considered for the clinical treatment of obesity. Given that fat reduction is known to extend lifespan in mice [25], these studies provided a potential mechanistic explanation for resveratrol-mediated increases in lifespan observed in every model organism tested to date.

Newly discovered pathways are now being targeted for therapeutic clinical obesity drug development. Knockdown of zebrafish GPR142a causes a significant decrease in detectable fat without causing overt developmental defects (Fig. 6), thus supporting the notion that this may be a good target for the development of anti-obesity drugs (Fig. 6). Little is known regarding GPR142. The first published report of GPR142 based purely on genomic sequence mentioned its presence in zebrafish. GPR142 is expressed throughout the brain as well as spleen, liver, kidney, and testes [26]. No examination of adipose tissue expression has been reported to date. GPR142 is an orphan G-PCR – neither endogenous nor xenobiotic ligands are known. Given the high level of GPR142 expression in so many tissues and its drug accessibility as receptor, this would appear to be an ideal anti-obesity drug target. More GPR142 research is needed to determine whether it is expressed in adipose tissue and what the effect of GPR142 antagonism may have on fat content in mammals along with possible toxicity.

While it was commonly believed that hormone sensitive lipase is the rate-limiting enzyme in lipolysis, the recent discovery of triglyceride lipases revealed a more complex picture [27]. Hormone sensitive lipase turns out to be the major lipase for catecholamine and natriuretic peptide-stimulated lipolysis, whereas adipose triglyceride lipase mediates basal triglyceride lipolysis [28]. What more this basic mechanism of lipolysis involving triglyceride lipase followed by hormone sensitive lipase and lastly monoglyceride lipase is conserved from yeast to man but is poorly understood [29].

Our experiments reveal for the first time that the direct addition of NAD causes a decrease in total fat content (Fig. 5A). These results are consistent with observations of the CD38 knockout mouse, which leads to a five-fold increase in NAD levels that results in prevention of high fat diet induced obesity through increased metabolic activity [30]. Along these lines, drugs targeting CD38 inhibition or NAD supplementation are only just recently being considered in obesity research. In one of the few examples examining the direct application NAD to an animal, intranasal administration of NAD was determined to confer great protection from transient focal ischemia in rats. This was quantified by infarct size measured in the brain hemisphere respectively connected with the given nostril where the negative control was the untreated opposite hemisphere [31]. The whole zebrafish nile red fluorescence fat metabolism assay can help narrow the list of potential future drug targets for treating clinical obesity.

## Competing interests

The authors declare that they have no competing interests.

## Authors' contributions

WTP developed the original conceptual framework for the study, performed most of the experiments, and prepared the manuscript. KSJ performed initial experiments developing the zebrafish-based assay. LAW measured cholesterol. RJJ measured total phospholipids and helped prepare the manuscript. APA and HLR performed quantitative real-time PCR. All authors read and approved the final version.
